# Mesotrione alters the structure of network interactions between soil microbes and affects C and N cycling functions

**DOI:** 10.3389/fmicb.2025.1708771

**Published:** 2026-01-02

**Authors:** Xiaojun Wu, Ruicao Feng, Liuti Cai, Ning Lu, Musen Lin, Gui Gao, Ying Wang, You Peng, Hancheng Wang

**Affiliations:** 1 College of Agriculture, Guizhou University, Guiyang, Guizhou, China; 2 Hubei Engineering Research Center for Pest Forewarning and Management, Yangtze University, Jingzhou, Hubei, China; 3 Guizhou Provincial Academician Workstation of Microbiology and Health, Guizhou Academy of Tobacco Science, Guiyang, Guizhou, China; 4 Zunyi Tobacco Company, Zunyi, Guizhou, China; 5 Qianxinan Municipal Company of Guizhou Tobacco Company, Qianxinan, Guizhou, China; 6 Anshun Company of Guizhou Tobacco Company, Anshun, Guizhou, China; 7 Xishui Branch of Zunyi Tobacco Company, Zunyi, Guizhou, China

**Keywords:** mesotrione, microorganism composition, soil carbon and nitrogen cycling, functionalgene, network interactions, Biolog-ECO microplate

## Abstract

Mesotrione is a widely used herbicide in corn production. However, its persistence in soil following application can cause significant damage to subsequent crops such as tobacco. Despite its widespread use, it remains unclear how mesotrione applications affect soil carbon and nitrogen cycling, as well as whether they alter soil microbial communities. Here, we conducted a 2-month greenhouse experiment to investigate changes in functional C and N cycling genes as well as the structural assembly of soil microbial communities following mesotrione application at 180 (T1) and 900 (T5) g a.i. ha^−1^. The results showed that total nitrogen, available nitrogen and organic matter in the soil were significantly reduced at both 30 and 60 days after mesotrione application (T1 and T5) compared to untreated control. Mesotrione significantly reduced the *α*-diversity of soil microbial communities at 10 days, and suppressed metabolic activity across multiple carbon sources over extended periods. The structure of the soil microbial exhibited complex dynamics from 1 to 60 days after mesotrione application, with a significant increase in the relative abundance of *Nocardioides* observed specifically in the T5 treatment. The effects of mesotrione on functional genes mediating the same C-cycling processes (carbon fixation pathway and methane metabolism) were differential. Mesotrione exhibited inhibitory effects on functional genes involved in nitrification, denitrification, and assimilatory nitrate reduction processes within the soil N-cycling, as well as promoting effects on those mediating nitrogen fixation. Network analyses revealed that soil microbial communities exhibited greater complexity and higher connectivity under high concentrations of mesotrione stress, with low abundance microbial genera forming a highly connected modular center. The number of microbial genera associated with key functional genes involved in soil C and N cycling increased following mesotrione application. Taken together, our findings provide theoretical insights into the microbial mechanisms underlying mesotrione’s impacts on soil carbon and nitrogen cycling.

## Introduction

Mesotrione is an inhibitor of 4-hydroxyphenylpyruvate dioxygenase (HPPD) and functions as a herbicide. It is characterized as a highly effective, low-toxicity and broad-spectrum selective herbicide, widely used for the control of broad-leaved weeds in corn fields (Gao et al., 2022; Li et al., 2021; Li et al., 2022). Currently, 169 commercial products containing mesotrione as the active ingredient have been registered in China (China Pesticide Information Network, 2023). Mesotrione eventually reaches the soil through various pathways, with half-lives ranging from 6 to 34.7 days in field soils, which vary considerably across different soil types (Quan et al., 2015; Wang et al., 2015). In recent years, with the increasing application of mesotrione, significant damage has been reported in certain non-target crops due to residual mesotrione in the soil. Twelve months after application in corn fields, mesotrione residues were still found to cause damage to rotational crops such as cranberries, soybeans and kidney beans (Felix et al., 2007; Soltani et al., 2007). In addition, previous research from our laboratory group found that transplanting tobacco plants at 60 days after mesotrione treatment still resulted in severe damage (Supplementary Figure S1). Furthermore, the excessive use of chemical herbicides may pose potential risks to soil microorganisms. Zheng et al. (2013) investigated the effects of organochlorine pesticides (OCPs) on soil microbial communities using the Biolog-ECO microplate, and found that high application levels of organochlorine pesticides significantly reduced the overall metabolic activity of soil microbes in utilizing carbon sources. Furthermore, the metabolism of amine carbon sources was suppressed, whereas that of phenolic compound carbon sources was enhanced. Wang et al. (2020) reported that flusulfuron-methyl could influence major microbial functional groups in sugarcane rhizosphere soil, including those involved in nitrite oxidation, photoautotrophic and phototrophic processes. Du et al. (2018) demonstrated that mesotrione treatment induced changes in both the structure and diversity of soil microbial communities and reduced the abundance of nitrogen-cycling-related genes, including *AOA-amoA* and *AOB-amoA.* Sun et al. (2016) investigated the ecological effects of mesotrione on soil microorganisms and found that a threshold dose of mesotrione could stimulate soil microbial activity, enhance soil microbial diversity and richness, and promote carbon source metabolic activity within microbial communities.

Microbes serve as key indicators of soil microenvironment health, where the interactions among species and functions within the soil microbial community drive material cycling (including C and N cycles), maintaining a dynamic equilibrium (Zhong et al., 2018). As essential elements of biogeochemical cycles, carbon and nitrogen play critical roles in ecosystem functioning, with their respective cycles-C (including carbon fixation, methane metabolism, and carbon degradation) and N (encompassing nitrogen fixation, ammonification, nitrification and denitrification) being closely linked to soil microbial activities (Kuypers et al., 2018). In recent years, with the development of high-throughput sequencing technology, the prediction of soil microbial carbon and nitrogen cycling from the perspective of microbial functional gene abundance has attracted widespread attention. Studies have shown that genes such as *cbbL*, *porA*, *oorA* and *accA* are involved in the carbon fixation pathway; *mcrA* and *pmoA* participate in methane metabolism; *nifH* is associated with nitrogen fixation pathway; *nxrA* plays a role in nitrification; *nirK*, *nirS* and *nosZ* are involved in denitrification; and *nirA* is linked to the assimilatory nitrate-reducing pathway (Kou-Giesbrecht et al., 2023; Buchkowski et al., 2015).

Excessive application of chemical herbicides can alter the structure and diversity of soil microbial communities as well as the abundance of functional genes, thereby indirectly disrupting the microbial-mediated biochemical cycling of carbon and nitrogen in soil (Cao et al., 2023). Members of the soil microbial community are not isolated from each other, and the interactions among species clusters and functional genes drive a series of biochemical cycling processes (Freilich et al., 2010; Fuhrman, 2009). Investigating interspecies interactions (i.e., co-occurrence networks) within complex microbial communities can contribute to a deeper understanding of potential interaction patterns among microbial taxa, regulatory characteristics of functional interactions, and the ecofunctional roles of rare species. Rong et al. (2021) reported that clomazone influences carbon and nitrogen cycling functions in soil ecosystems by altering the interspecies interactions within soil microbial communities, which tend to evolve toward more complex and highly connected network under clomazone stress. Cao et al. (2023) revealed that mesosulfuron-methyl stress altered the structure of soil microbial communities. The microbial taxa associated with nitrogen cycling genes exhibited significant changes following exposure to mesotrione, and the complexity of co-occurrence networks among these taxa decreased, leading to the formation of multiple networks modules that were negatively correlated with nitrogen cycling functional genes. Mesotrione is mainly biodegraded in soil (Crouzet et al., 2010). However, the microbial degradation pathway of mesotrione in soil remains incompletely understood. Previous studies investigating the effects of mesotrione on soil microorganisms were primarily focused on single-factor analyses of its impacts on soil microbial community structure, diversity, and functional characteristics (Crouzet et al., 2016). Under the stress of mesotrione herbicide, the interactions among soil microbial species groups and the interactions between microorganisms and genes related to soil carbon and nitrogen cycles remain unclear.

Therefore, in this study, the Biolog-ECO microplate technology was adopted to assess the changes in the metabolic functional diversity of soil microbial communities after mesotrione treatment. This study employed metagenomic sequencing technology to deeply investigate the changes in the core microbiota of soil after the application of mesotrione, as well as the diversity and abundance variations of functional genes involved in carbon and nitrogen cycling. To elucidate the response mechanisms of the soil microbial community under mesotrione stress, a network interaction model was constructed between microbial groups and functional attributes. Our findings provide a theoretical basis for evaluating the ecological safety of mesotrione in soil ecosystems.

## Materials and methods

### Experimental design and soil sample collection

This experiment was conducted in a greenhouse at the Tobacco Green Control Base (N24°38′, E104°51′) in Xingyi City, Guizhou Province, China. The experimental greenhouses were equipped with insulation, movable shading systems, and irrigation facilities. The soil at the test site was a yellow loam, and watermelon seedlings were planted in the absence of herbicide application prior to the experiment. For the subsequent experiment, weeds were removed from the test plot, the soil was tilled and the surface was leveled over a 5 day period prior to spray treatment. A randomized block design was implemented with plot dimensions of 6 m^2^ (3 m × 2 m), and the treatment plots were separated by manually excavated trenches. On the day preceding the treatment, the test plots were irrigated to full soil saturation using automatic sprinkler systems. Mesotrione (40% mesotrione SC; manufactured by Zhongshan Chemistry Industry Co., Ltd.; Zhejiang, China) was applied on March 20, 2023, and three treatments levels were implemented: i (1) CK, water without mesotrione application; (2) T1, the recommended field application rate (180 g of active ingredient (a.i.) per hectare (hm^−2^)); and (3) T5, five times the recommended dose (900.00 g (a.i.) hm^−2^). Three replicate plots were assigned to each treatment. Mesotrione was dissolved in 3 liters of water and the solution was uniformly applied to the soil surface of the corresponding plots (totalling 6 m^2^) using a backpack automatic sprayer. The sprayer was thoroughly cleaned between successive treatments. The average temperature during the experiment ranged from 12.8 °C to 17.6 °C, and the soil was maintained at a moist condition through regular and controlled irrigation every 10 days.

Soil samples from the 0–20 cm depth layer were collected from each plot using five equidistant sampling points at 0 (before treatment), 1, 5, 10, 20, 30, and 60 days after mesotrione treatment. Sampling was conducted in the buffer zones between plots to prevent cross contamination among treatments. The five subsamples from each plot were mixed into one composite sample, resulting in a total of 63 samples (3 treatments × 3 replicates × 7 collections). After removing stones and plant debris, each sample was divided into three portions. The first portion was stored at −20 °C for analysis of microbial carbon source metabolic function. The second portion was slightly air-dried, sieved through a 2-millimeter sieve (each filter sieve was sterilized), and then rapidly stored at −80 °C for metagenomic analysis. The third portion was air-dried, sieved through a 2-millimeter sieve, and stored at −20 °C for determination of soil physicochemical properties and mesotrione residues.

### Determination of soil physicochemical properties

The soil physicochemical properties was investigated following the methods described in Soil and Agricultural Chemistry Analysis and Soil Physicochemical Analysis (Bao, 2005; Lu, 2000). Total nitrogen was determined using the Kjeldahl method, available nitrogen by the alkaline hydrolysis diffusion method, available phosphorus by the ammonium bicarbonate extraction-molybdenum antimony anti-colorimetric method (Olsen), available potassium by flame atomic absorption spectrometry, and organic matter content by the potassium dichromate-external heating method. Soil pH was measured using a calibrated pH meter.

### Effect of mesotrione on the metabolic capacity of soil microorganisms

The Biolog-ECO microplates (Biolog Inc., Hayward, CA, United States) contain a total of 31 carbon sources, which are widely utilized by microorganisms in natural environments (Rutgers et al., 2016). Changes in absorbance values of the Biolog-ECO microplate can be measured to indirectly reflect the metabolic capacity of microorganisms for carbon sources (Feng et al., 2023). For metabolic capacity test, the soil samples stored at −20 °C freezer were taken out and pre-incubated in a greenhouse at 25 °C for 24 h. Ten grams of each sample was transferred into a 200 mL Erlenmeyer flask containing 100 mL of 0.85% sterilized saline. The flasks were incubated at 28 °C with shaking at 180 r/min for 2 h, followed by a 1-h resting period (Li et al., 2021; Li et al., 2022). One milliliter of the supernatant was aspirated and diluted a hundred-fold dilution, and subsequently transferred to the Biolog-ECO microplate for carbon source metabolic analysis using an 8-channel motorized pipette at a volume of 100 μL per well. After inoculation, the Biolog-ECO microplates were placed in the OmniLog incubator and incubated at a constant temperature of 28 °C. The Biolog D5E_OKA_data.exe software was used to capture color changes in metabolic wells during incubation. The carbon source metabolic activity of soil microorganisms was analyzed by using Heml software (Feng et al., 2023). The metabolic color change curve reached a plateau after 96 h of incubation, indicating that microbial metabolic activity had stabilized. The metabolic functions of soil microorganisms were subsequently analyzed based on the OmniLog readings obtained at the 96-h time point. Functional diversity was further assessed using the Simpson diversity index (D), Shannon–Wiener diversity index (H′), and McIntosh index (U) (Zhao et al., 2020).

### DNA extraction and metagenomic sequencing

A total of 0.5 g of each dried soil sample was used for soil microorganism DNA extraction using the Magnetic Soil and Stool DNA Kit (TianGen Biotech Co., Ltd., China). The concentration and purity of DNA in the extracted samples were detected using NanoDrop. The genomic DNA was randomly fragmented into segments of appropriate length, and then a library was constructed through end repair, adapter ligation and PCR amplification. Metagenomic libraries were size selected to yield fragments of approximately 350 bp. These libraries were then subjected to quality assurance and sequenced on an Illumina PE150 platform at Novogene Bioinformatics Technology Co., Ltd. (Beijing, China).

### Analysis of metagenomic data

The Readfq software was utilized to perform quality control on the raw sequencing data, resulting in the generation of clean data (Karlsson et al., 2013). The cleaned data were assembled and analyzed using the Megahit software. Subsequently, the assembled scaffolds were fractured to obtain the sequence of scaffold fragments (Li et al., 2015; Qin et al., 2014). MetaGeneMark was used for ORF prediction based on scaftigs of individual samples (≥500 bp). The predicted open reading frames were subsequently processed to remove redundancy, resulting in the construction of non-redundant gene catalogues (Karlsson et al., 2012; Li et al., 2014). Starting with the gene catalogue, the Clean Data from each sample was integrated to determine the abundance of genes within individual samples. The Unigenes were annotated against the MicroNR database using Diamond software. Combine the results with the gene abundance table to obtain the species abundance information at different classification levels (Buchfink et al., 2015; Huson et al., 2011; Feng et al., 2015). Unigenes were annotated by performing sequence alignment against the Kyoto Encyclopedia of Genes and Genomes (KEGG, V.2023.02) and the Carbohydrate-Active enzymes (CAZy, V.2023.03) databases using DIAMOND software (blastp algorithm, e-value ≤ 1e-5), under stringent criteria to ensure functional annotation accuracy. The relative abundance of the different functional categories was calculated (Kanehisa et al., 2017; Kanehisa et al., 2006; Cantarel et al., 2009).

### Statistical analyses

Correlation analysis was performed based on the species abundance tables and the functional abundance tables. The *α*- diversity index, including the Shannon index and Chao1 index, of soil microbial communities was calculated using Mothur software (Version 1.30.2). The *β* diversity of soil microbial communities were analyzed with Principal Component Analysis (PCA) using Euclidean distances in the R software (Version 4.0.3) ‘Vegan’ package. Differences in diversity indices-including Shannon index, Chao1 index, Average Well Color Development (AWCD), Simpson diversity index (D), Shannon -Wiener diversity index (H′), and McIntosh index (U)-as well as carbon-cycle gene abundances (*mcrA, pmoA, cbbL, porA, accA,* and *oorA*) and N-cycle gene abundances (*nirA, nirK, nosZ, nxrA, nirS,* and *nifH*) across treatments were statistically analyzed using one-way ANOVA in SPSS 25.0 (Kou-Giesbrecht et al., 2023; Dahal et al., 2017; Buchkowski et al., 2015).

To investigate the effects of mesotrione on the soil microbial community and the potential relationships between microbial taxa and functional genes, co-occurrence patterns were constructed among soil microbial species, as well as between microbes and functional genes. The top 300 most relatively abundant genera were selected for the construction of the microbial interactions network (Rong et al., 2021). Based on a comprehensive literature review, we identified key functional genes associated with carbon and nitrogen cycling processes and selected the 30 most abundant microbial phyla to construct a microbial-functional gene interaction network (Wang et al., 2023). When the Spearman correlation coefficient (R) was > 0.9 and the *p*-value was <0.05, it indicates that the Spearman correlation between the two genera was statistically significant. Spearman’s correlation between functional genes and species was considered statistically significant when the Spearman correlation coefficient (R) > 0.6 and the p-value was < 0.05 (Chen et al., 2019). Networks were visualized using Gephi software (version 0.10.1) (Bastian et al., 2009).

## Results

### Effect of mesotrione on the physicochemical properties in soil

The soil physicochemical properties were shown in Table 1. Among them, the available phosphorus content in the T1 treatment was significantly higher than that in the control treatment (*p* < 0.05). Compared with the control, the total nitrogen, available nitrogen, and organic matter contents in the T1 and T5 treatments were significantly reduced at both 30 and 60 days post-treatment (*p* < 0.05). The available phosphorus in the T1 and T5 treatments remained significantly higher than that in the control at both 30 and 60 days (*p* < 0.05). On the 30 and 60 days, the pH values of the T1 treatment were significantly higher than those of the control group (*p* < 0.05). Furthermore, the available potassium in the T1 treatment was significantly lower than that in the control group at 30 days (*p* < 0.05), whereas in the T5 treatment, the available potassium was significantly reduced compared to the control group at both 30 and 60 days (*p* < 0.05).

**Table 1:** Effect of mesotrione on soil physicochemical properties.

Sampling time	Grouping of samples	TN	AN	AP	AK	SOM	pH
		N(%)	N(mg/kg)	P(mg/kg)	K(mg/kg)	g/kg	
0d	CK	0.22 ± 0.00a	86.83 ± 2.47a	70.67 ± 0.41b	675.33 ± 4.37b	34.13 ± 0.67a	7.00 ± 0.00a
	T1	0.22 ± 0.00a	91.43 ± 1.37a	72.60 ± 0.06a	688.00 ± 3.46a	34.67 ± 0.43a	7.00 ± 0.00a
	T5	0.22 ± 0.00a	88.67 ± 3.12a	71.07 ± 0.27b	685.33 ± 4.67a	35.13 ± 0.97a	7.07 ± 0.03a
30d	CK	0.22 ± 0.00a	87.53 ± 1.63a	70.77 ± 0.72c	683.33 ± 5.81a	35.40 ± 1.12a	7.03 ± 0.03b
	T1	0.17 ± 0.00b	65.10 ± 0.81b	86.90 ± 0.81a	597.33 ± 1.76b	25.20 ± 0.25b	7.33 ± 0.03a
	T5	0.16 ± 0.00c	66.03 ± 0.93b	78.33 ± 0.28b	620.00 ± 11.02b	24.27 ± 0.03b	7.13 ± 0.03b
60d	CK	0.22 ± 0.00a	89.60 ± 3.41a	71.63 ± 0.32c	684.67 ± 1.76a	35.13 ± 0.85a	7.10 ± 0.00b
	T1	0.16 ± 0.00b	64.63 ± 0.47b	87.43 ± 0.30a	676.67 ± 2.40a	25.20 ± 0.25b	7.30 ± 0.00a
	T5	0.16 ± 0.00b	64.63 ± 1.23b	74.90 ± 0.40b	572.67 ± 4.67b	24.47 ± 0.22b	7.13 ± 0.07b

Data followed with lower letters in each column are separated by one-way ANOVA (*p* < 0.05). Mean values (*n* = 3) ± SE. CK no mesotrione, T1: 180.00 g a.i. hm^−2^, T5: 900.00 g a.i. hm^−2^, TN: total nitrogen, AN: available nitrogen, AP: available phosphorus, AK: available potassium, SOM: soil organic matter.

### Effects of mesotrione on the diversity of metabolic functions of soil microbial community

**Figure 1:**
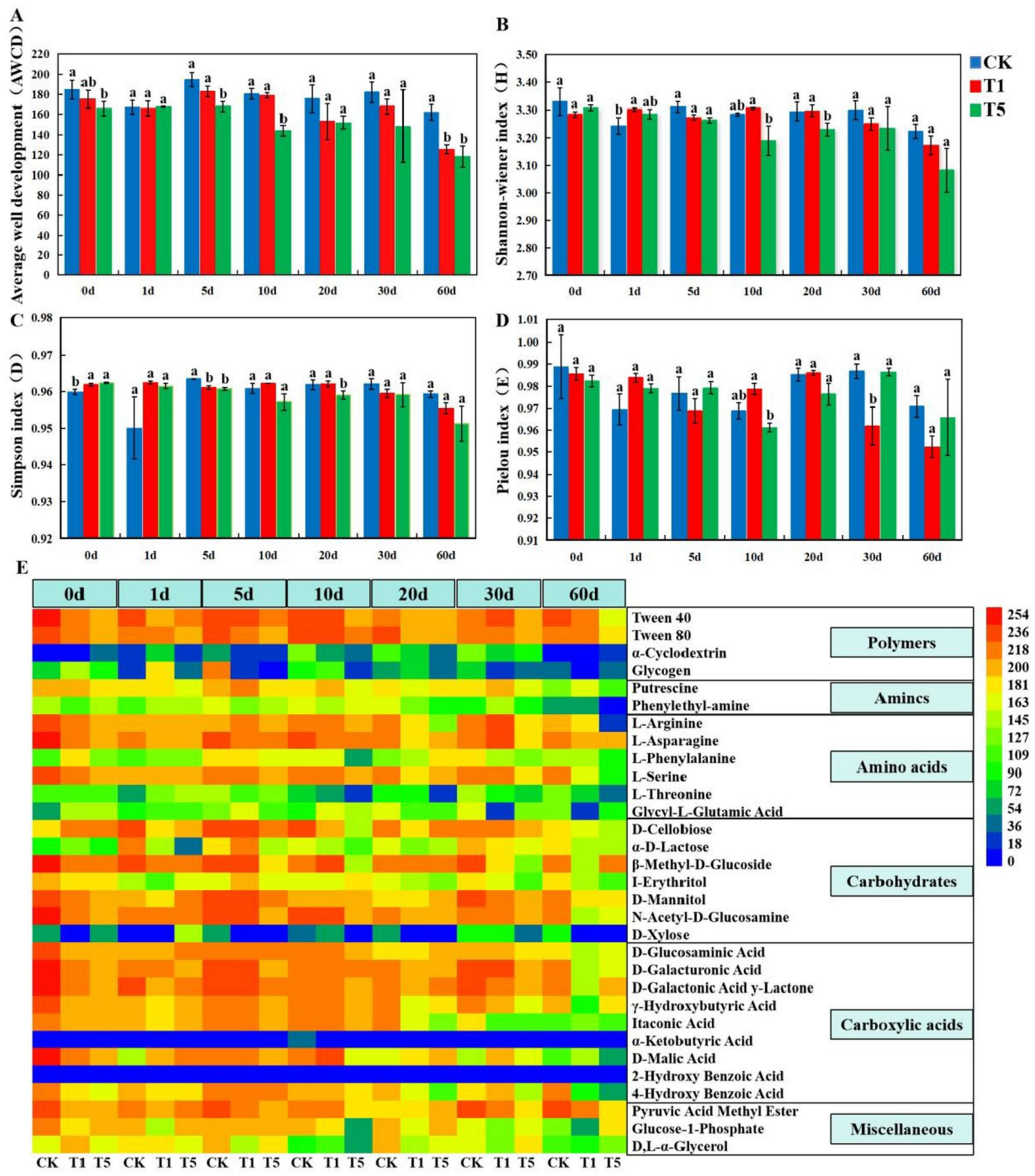
Effect of mesotrione on carbon source metabolism function in soil microorganisms. **(A)** Average well color development (AWCD); **(B)** Shannon-Wiener diversity index (H′); **(C)** Simpson diversity index (D); **(D)** McIntosh index (U); **(E)** Clustered heat map of soil microbial carbon source metabolism. Lowercase letters (a–c) denote statistically significant differences among different treatments at the same sampling time based on one-way ANOVA (*p* < 0.05). Mean values (*n* = 3) ± SE. CK no mesotrione, T1: 180.00 g a.i. hm^−2^, T5: 900.00 g a.i. hm^−2^.

Compared with the control group, the overall metabolic activity (AWCD) of soil microorganisms toward carbon sources was reduced in the mesotrione-treated (T1 and T5). This reduction was statistically significant (*p* < 0.05) in the T5 treatment at 5, 10, and 60 days, as well as in the T1 treatment at 60 days (Figure 1A). The Shannon index in the T1 treatment was significantly increased at 1 days (*p* < 0.05), whereas that in the T5 treatment was significantly decreased at 10 and 20 days (*p* < 0.05) (Figure 1B). The Simpson index in the T1 treatment was significantly lower at 5 days (*p* < 0.05), whereas that in the T5 treatment was significantly lower at 5 and 20 days (*p* < 0.05) (Figure 1C). The McIntosh index in the T1 treatment was significantly lower at 30 days compared to the control (*p* < 0.05) (Figure 1D). The metabolic activity of soil microorganisms toward different carbon sources vary. Among them, the metabolic activities associated with N-Acetyl-D-Glucosamine, Tween 40, Tween 80, L-Arginine, D-Galacturonic Acid, L-Asparagine, L-Serine, and D-Mannitol were relatively high. In contrast, the metabolic activities of *α*-Cyclodextrin, Glycogen, D-Xylose, Phenylethyl-amine, L-Phenylalanine, L-Threonine, Glycyl-L-Glutamic Acid and I-Erythritol were relatively low and α-Ketobutyric Acid, 2-Hydroxy Benzoic Acid cannot be metabolized by the microbial community (Figure 1E). After treatment with mesotrione, the metabolic activity of soil microorganisms towards most carbon sources decreased over time. Among them, the metabolic activities of soil microorganisms in the T5 treatment associated with Tween 40, Tween 80, and L-Arginine were reduced at 60 days. The metabolic activities of soil microorganisms in the T1 treatment for *β*-Methyl-D- Glucoside, D-Galactonic Acid y-Lactone, and *γ*-Hydroxybutyric Acid metabolic activity decreased at 60 days. The metabolic activity of soil microorganisms in the T1 and T5 treatments for D-Glucosaminic Acid, D-Galacturonic Acid, L-Serine and 4-Hydroxy Benzoic Acid all decreased at 60 days.

### Effects of mesotrione on the structure of soil microbial community

**Figure 2:**
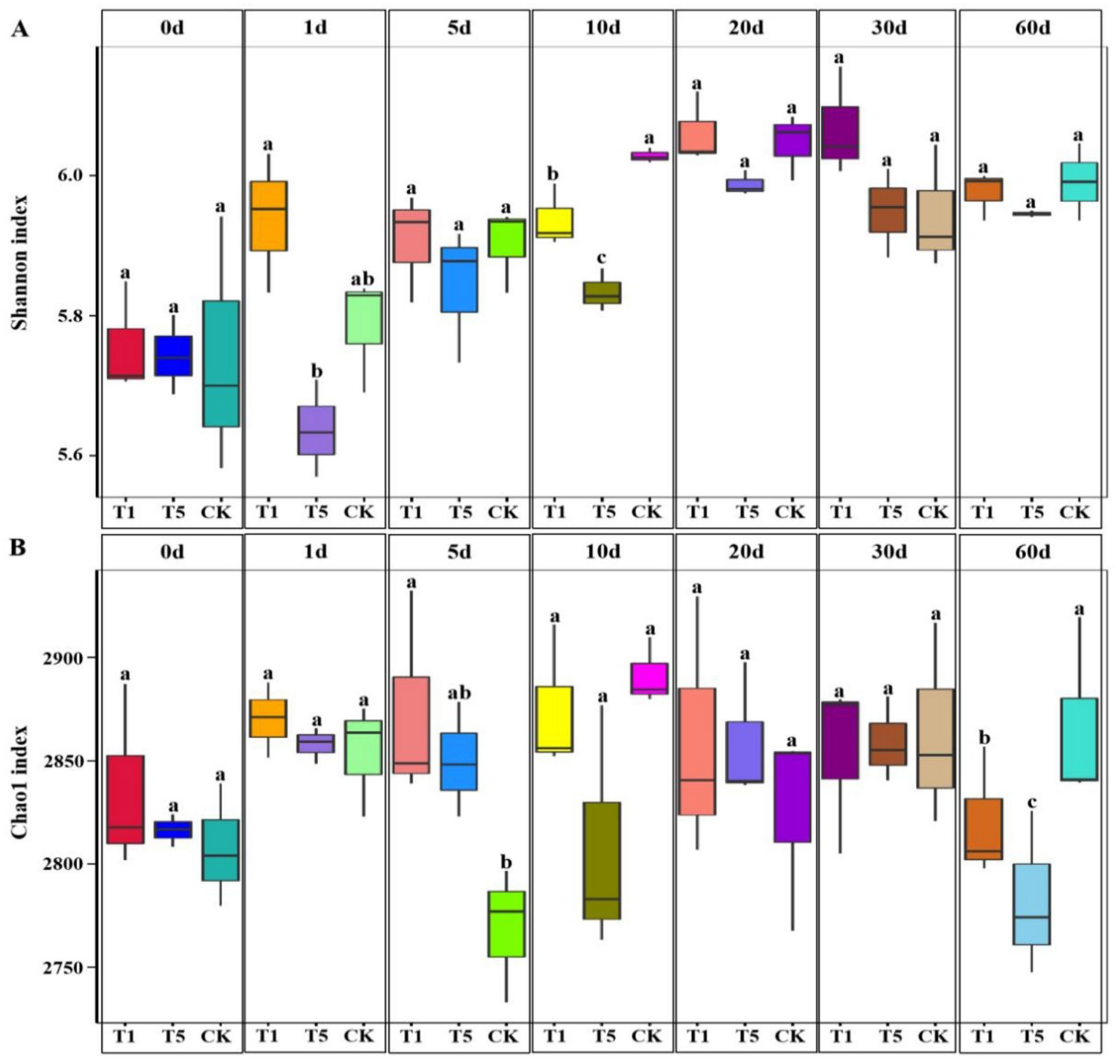
Box plot of the effect of mesotrione on the *α*-diversity (**A**: Shannon index, **B**: Chao1 index) of soil microbial communities. Different letters represent significant differences between treatments (*p* < 0.05). CK no mesotrione, T1: 180.00 g a.i. hm^−2^, T5: 900.00 g a.i. hm^−2^.

**Figure 3:**
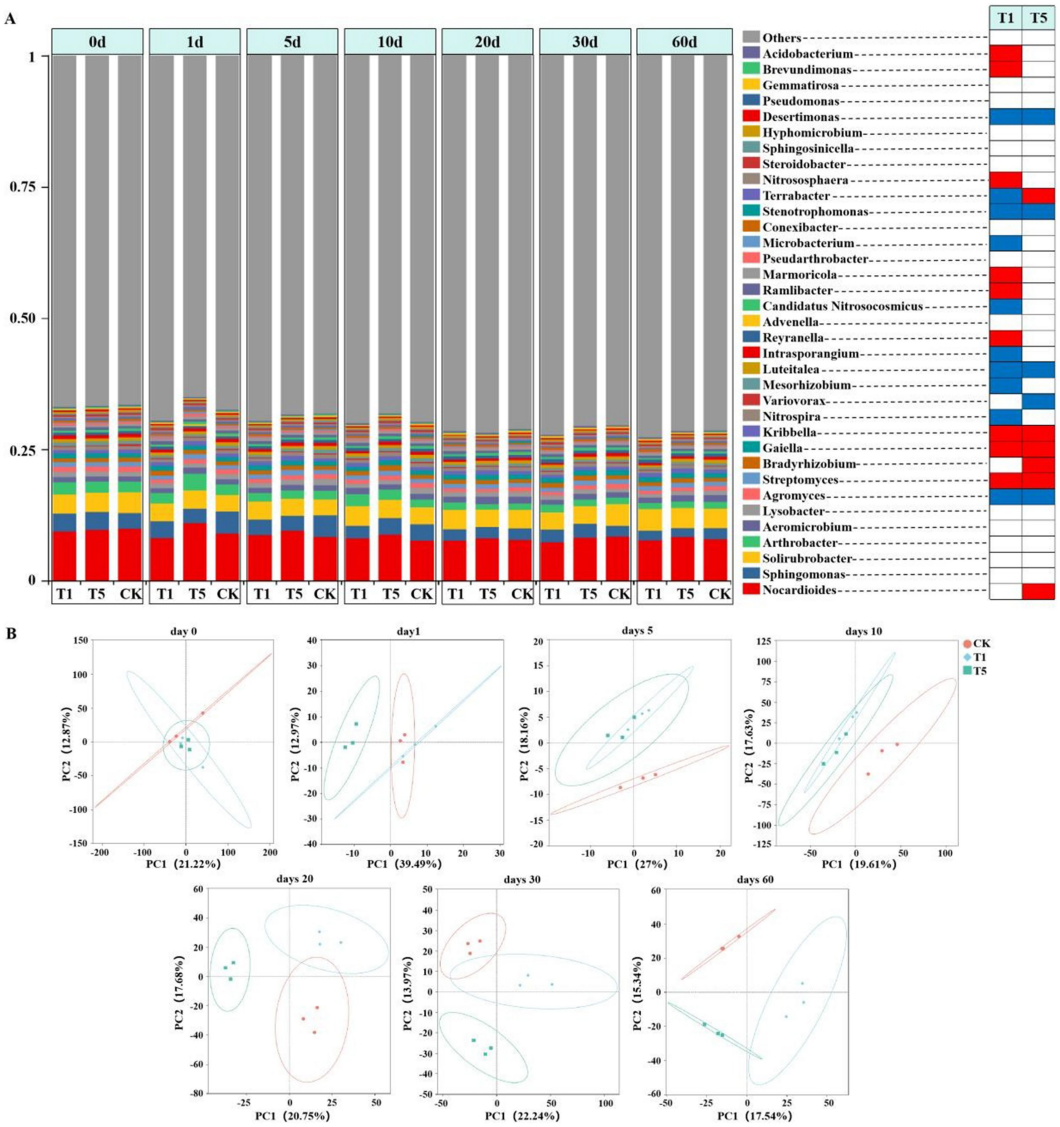
Effects of mesotrione on soil microbial composition and community structure. The relative abundance of soil microorganisms (at the genus level, top 35) in different treatments at different sampling periods was shown in Figure **(A)**, and genera that showed a significant (*p* < 0.05) increased relative abundance after mesotrione-treated (red) or control (green) treatments were shown. **(B)** PCoA of the soil microbial community based on Bray-Curtis distances on days 0, 1, 5, 10, 20, 30, and 60. CK no mesotrione, T1: 180.00 g a.i. hm^−2^, T5: 900.00 g a.i. hm^−2^.

The composition and diversity of soil microbial communities were analyzed using metagenomics sequencing. A total of 413,087 clean data were obtained, among which the percentages of Effective, Clean_Q20, and Clean_Q30 were 99.48, 96.28, and 91.31%, respectively. Metagenomics sequencing results revealed that bacteria, archaea, eukaryota, viruses and other microorganisms accounted for 92.84, 1.26, 0.03, 0.01 and 5.94% of the microorganisms in the soil samples, respectively. Compared with the control, the Shannon index of the soil microbial community was significantly reduced in the T1 and T5 treatments at 10 days but showed recovery at 20 days (*p* < 0.05) (Figure 2A). The richness (Chao1 index) of the soil microbial community was significantly reduced in the T1 and T5 treatments at 60 days (*p* < 0.05) (Figure 2B). At the phyla level, the dominant phyla across all treatments were Actinomycetota, Pseudomonadota, Thermomicrobiota, Gemmatimonadota Acidobacteriota, Chloroflexota, Nitrososphaerota, and Planctomycetota (Supplementary Figure S1). At the genus level, the relative abundance of *Streptomyces*, *Gaiella* and *Kribbella* were significantly increased in all mesotrione treatment groups (T1 and T5) compared to the control (*p* < 0.05). In contrast, the relative abundance of *Agromyces*, *Luteitalea*, *Stenotrophomonas*, and *Desertimonas* were significantly (*p* < 0.05) decreased. Compared to the control, the relative abundance of *Reyranella*, *Ramlibacter*, *Marmoricola*, *Nitrososphaera*, *Brevundimonas*, and *Acidobacterium* were significantly increased in the T1 treatment (*p* < 0.05), whereas those of *Nitrospira*, *Candidatus Nitrosocosmicus*, *Mesorhizobium*, *Intrasporangium*, *Microbacterium* and *Terrabacter* were significantly decreased (*p* < 0.05). Furthermore, the relative abundance of *Nocardioides*, *Bradyrhizobium*, and *Terrabacter* were significantly increased (*p* < 0.05), while those of Var*iovorax* and *Desertimonas* were significantly decreased in the T5 treatment (*p* < 0.05) (Figure 3A). Principal Component Analysis (PCA) was employed to assess the effect of mesotrione treatment on soil microbial community structure (Figure 3B). After treatment with mesotrione, the soil microbial community in the T1 and T5 treatments exhibited complex dynamic changes across different time points. The soil microbial community structures of all treatments (T1 and T5) were different from that of the control. Notably, the soil microbial community distribution in the T5 treatment remained distinct from the control throughout the experimental period (1–60 days). In contrast, the soil microbial community distribution in the T1 treatment was separated from the control from 5 to 10 days post-treatment, but gradually recovered toward control levels by 20 days post-treatment as time progressed.

### Effect of mesotrione on co-occurrence networks between soil microbiomes

The impact of mesotrione on the interaction within the soil microbial community was investigated through the construction of a microbial co-occurrence network. Compared with the control, the total number of nodes, edges and average degree (avgK) was reduced in the T1 treatment. In contrast, the T5 treatment exhibited an increased total number of edges and a higher average degree (avgK) compared to the control group. Furthermore, the average path length, positive correlation ratio (%) and Network diameter in both the T1 and T5 treatments were lower than those observed in the control group, indicating that soil microorganisms developed more complex and highly interconnected co-occurring networks under mesotrione stress (Table 2).

**Table 2:** Topological indices of each network in Figure 4.

Network indexes	CK	T1	T5
Number of nodes	104	95	101
Number of edges	299	157	301
Positive correlation ratio (%)	83.61	81.53	53.23
negative correlation ratio (%)	16.39	18.47	46.77
Network diameter	9	8	5
Average path length	3.11	2.95	2.037
Average degree	5.75	3.305	6.139

**Figure 4:**
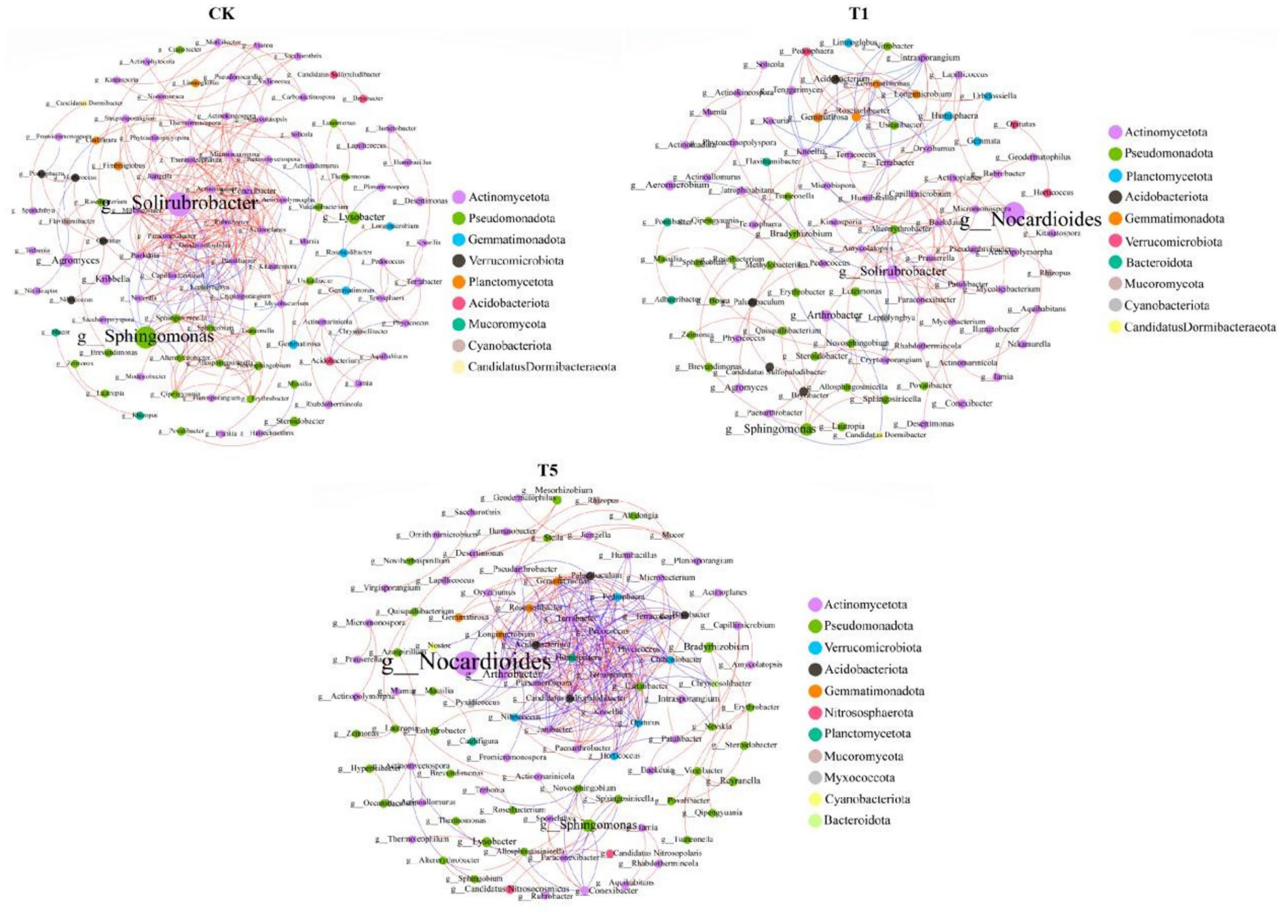
Network co-occurrence analysis of soil microbial communities of different treatments. A connection stands for a strong (Spearman’s *r* > 0.9) and significant (*p* < 0.05) correlation for the no treatment (CK) and mesotrione-treatments (T1 and T5). The size of each node is proportional to its relative abundance (degree). Red edges indicate positive interactions between two species genus nodes, while blue edges indicate negative interactions. Each node is colored based on the microbial phyla.

The co-occurrence network diagram of soil microorganisms revealed variations among the top 300 genera exhibiting significant correlations under different treatment conditions (Figure 4). In the control treatment, the genus with the highest relative abundance was *Solirubrobacter*, followed by *Sphingomonas* and *Lysobacter*. All high-connectivity genera belonged to Actinomycetota phylum. In both the T1 and T5 treatments, *Nocardioides* was the most abundant genus, followed by *Sphingomonas*. Notably, no high-connectivity genera were identified in the T1 treatment, whereas the high-connectivity genera in the T5 treatment belonged to the phyla Actinomycetota, Pseudomonadota, Acidobacteriota, Verrucomicrobiota, Planctomycetota, and Gemmatimonadota, respectively.

### Effects of mesotrione on the abundance of functional genes for C and N cycle in the soil microorganisms

**Figure 5:**
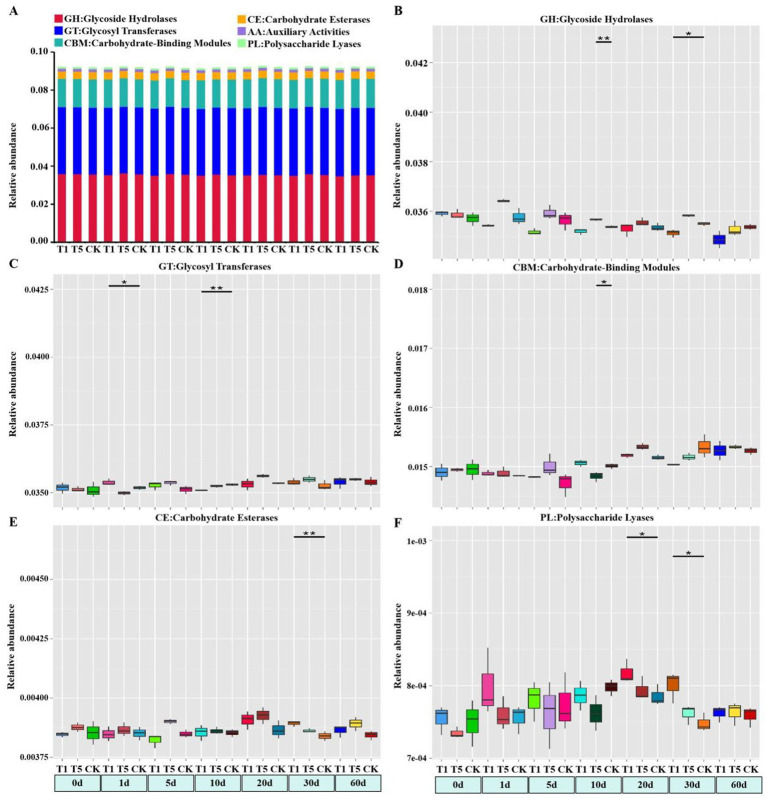
Effect of mesotrione on the relative abundance of soil microbial carbohydrate enzyme genes. **(A)** Histogram of relative abundance of carbohydrate enzyme genes in different treatments. Box plots **(B–F)** of the relative abundance of carbohydrate enzyme genes that were significantly (**p < 0.01, *p < 0.05) different in different treatments. CK no mesotrione, T1: 180.00 g a.i. hm^−2^, T5: 900.00 g a.i. hm^−2^.

In this study, metagenomics sequencing data were annotated using the CAZy database. Six carbohydrate-active enzyme gene families [Glycoside Hydrolases (GHs), Glycosyl Transferases (GTs), Polysaccharide Lyases (PLs), Carbohydrate Esterases (CEs), Auxiliary Activities (AAs) and Carbohydrate-Binding Modules (CBMs)] were annotated across all treatments in the CAZy database (Figure 5A). Among all the treatments, Glycoside Hydrolases (GHs) and Glycosyl Transferases (GTs) were the most dominant enzyme gene families, followed by Carbohydrate-Binding Modules (CBMs). There were different effects of mesotrione treatment on the six soil microbial carbohydrase genes. Notably, the abundance of Glycoside Hydrolases and Carbohydrate-Binding Modules in the T5 treatment was significantly higher than that in the control treatment at 10 days post-application (*p* < 0.05) (Figures 5B,D). For the T1 treatment, the abundance of Glycosyl Transferases (GTs) significantly increased (*p* < 0.05) at 1 day post-treatment, but subsequently significantly decreased at 10 days (*p* < 0.05) (Figure 5C). Compared to the control, the abundance of Carbohydrate Esterases (CEs) was significantly increased in the T1 treatment at 30 days (*p* < 0.05), and the abundance of Polysaccharide Lyases (PLs) was significantly increased in the T1 treatment at 20 and 30 days (*p* < 0.05) (Figures 5E,F).

**Figure 6:**
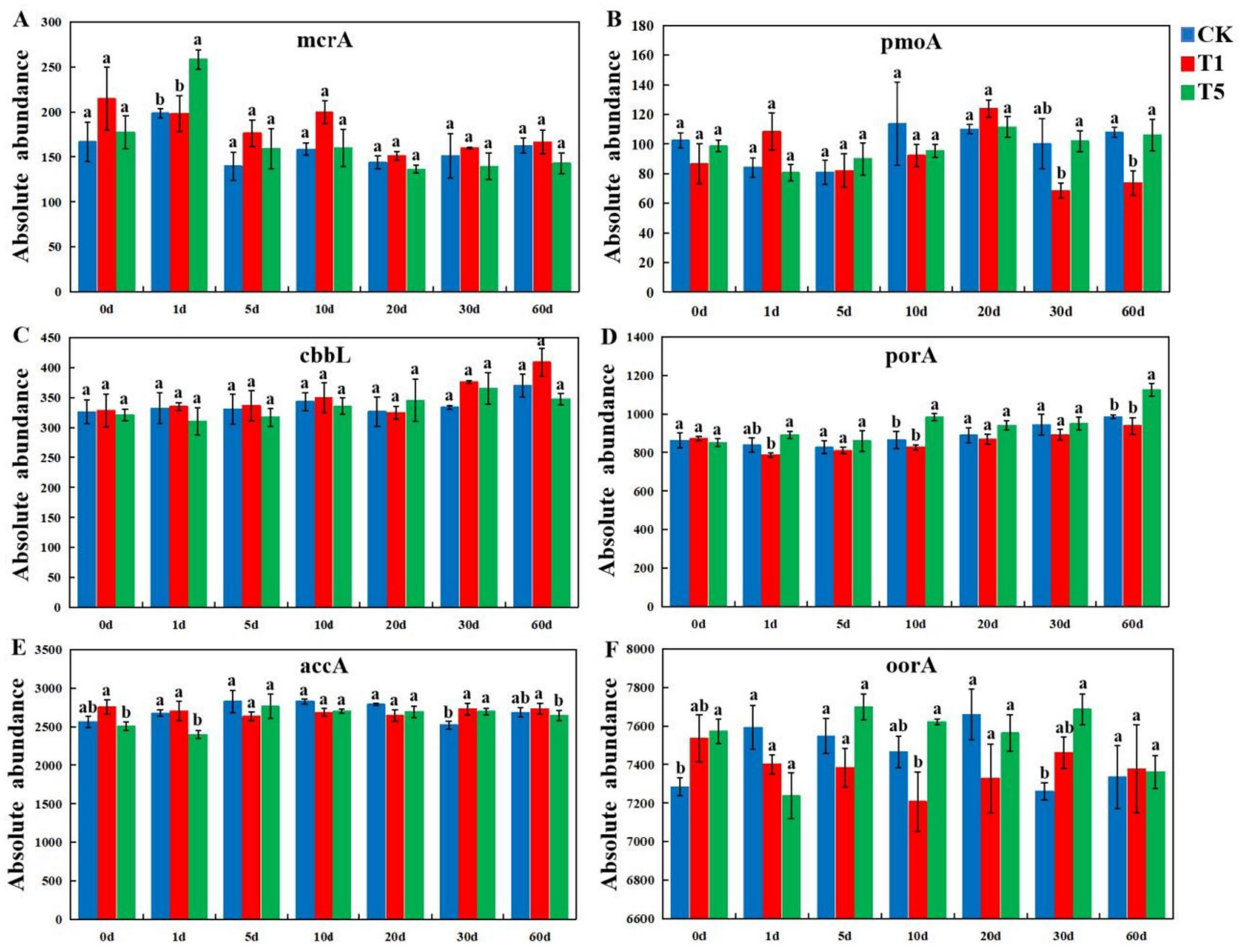
Effects of mesotrione (CK, T1, and T5) on the abundance of main C cycle genes [*mcrA* **(A)**, *pmoA* **(B)**, *cbbL* **(C)**, *porA* **(D)**, *accA* **(E)**, and *oorA* **(F)**]. Lowercase letters (a–c) indicate statistically significant differences among different treatments at the same sampling time using one-way ANOVA (*p* < 0.05). Mean values (*n = 3*) *± SE. CK n*o mesotrione, T1: 180.00 g a.i. hm^−2^, T5: 900.00 g a.i. hm^−2^.

**Figure 7:**
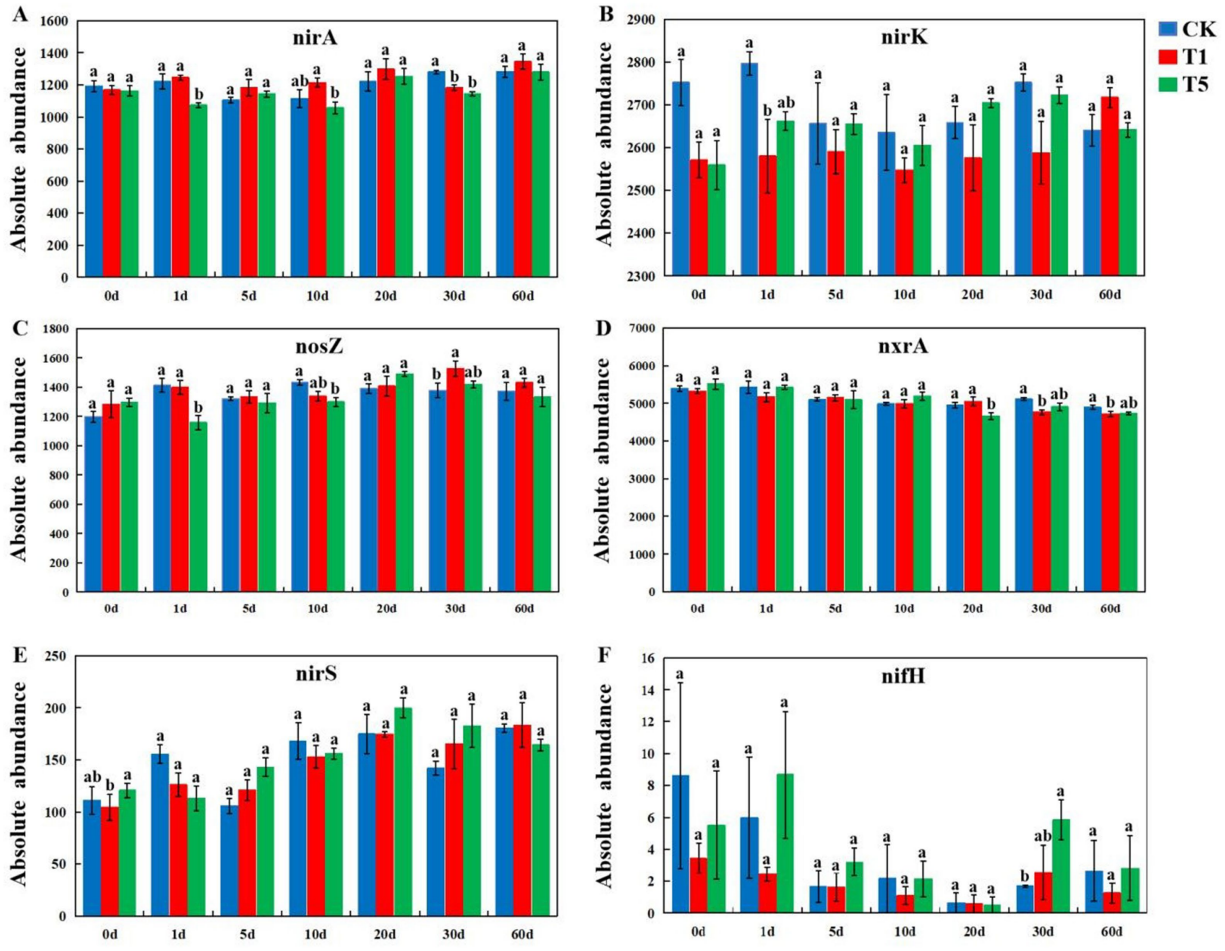
Effects of mesotrione (CK, T1 and T5) on the abundance of main N cycle genes [*nirA* **(A)**, *nirK* **(B)**, *nosZ* **(C)**, *nxrA* **(D)**, *nirS* **(E)**, and *nifH* **(F)**]. Lowercase letters (a–c) indicate statistically significant differences among different treatments at the same sampling time using one-way ANOVA (*p* < 0.05). Mean values (*n = 3*) *± SE. CK n*o mesotrione, T1: 180.00 g a.i. hm^−2^, T5: 900.00 g a.i. hm^−2^.

The metagenomics sequencing data were annotated using the KEGG functional database, and all treated soil microorganisms were classified into six metabolic pathway categories: Cellular processes, Environmental information processing, Genetic information processing, Human diseases, Metabolism, and Organismal systems (Supplementary Figure S2). The abundance of soil microbial metabolic pathway genes exhibited complex dynamics after mesotrione treatment. Compared to the control, the abundance of Metabolism and Environmental information processing genes increased, whereas the abundance of Cellular processes genes decreased in the T5 treatment at 1 days post-application. The abundance of genes associated with Genetic information processing, Human diseases, and Organismal systems declined in the T1 treatment across all time points (10, 20, 30, and 60 days post-application). In addition, this study further investigated the effects of mesotrione on key functional genes involved in the major carbon cycle (*mcrA*, *pmoA*, *cbbL*, *porA*, *accA* and *oorA*) and nitrogen cycle (*nirA*, *nirK*, *nosZ*, *nxrA*, *nirS* and *nifH*) pathways of soil microorganisms. In the C cycling pathway, the relative abundance of *pmoA* was significantly decreased (*p* < 0.05) at 60 days (Figure 6B), whereas that of *accA* was significantly increased (*p* < 0.05) at 30 days in the T1 treatment compared to the control (Figure 6E). For the T5 treatment, the relative abundance of *mcrA* was significantly increased (*p* < 0.05) at 1 days post-application (Figure 6A), and the relative abundance of *porA* was significantly increased (*p* < 0.05) at 10 and 60 days post-application (Figure 6D). The relative abundance of *accA* was significantly decreased (*p* < 0.05) at 1 days post-application but significantly increased (*p* < 0.05) at 30 days, whereas the relative abundance of *oorA* was significantly increased (*p* < 0.05) at 30 days post-application compared with the control (Figure 6F). The relative abundance of *cbbL* did not show significantly differences throughout the experiment period (1–60 days) (Figure 6C). In the N cycling pathway, for the T1 treatments, the relative abundance of *nirA* was significantly lower (*p* < 0.05) at 30 days post-application (Figure 7A); the relative abundance of *nirK* was significantly lower (*p* < 0.05) at 1 days post-application (Figure 7B). the relative abundance of *nosZ* was significantly higher (*p* < 0.05) at 30 days post-application (Figure 7C), and the relative abundance of *nxrA* was significantly lower (*p* < 0.05) at 30 and 60 days post-application compared to the control (Figure 7D). For the T5 treatment, the relative abundance of *nirA* was significantly lower (*p* < 0.05) at 1 and 30 days post-application, and the relative abundance of *nosZ* was significantly lower (*p* < 0.05) at 1 and 10 days post-application. The relative abundance of *nxrA* was significantly lower (*p* < 0.05) at 20 days post-application, and the relative abundance of *nifH* was significantly increased (*p* < 0.05) at 30 days post-application compared to the control (Figure 7F). The relative abundance of *nirS* did not show significant differences throughout the experimental period (1–60 days) (Figure 7E).

**Figure 8:**
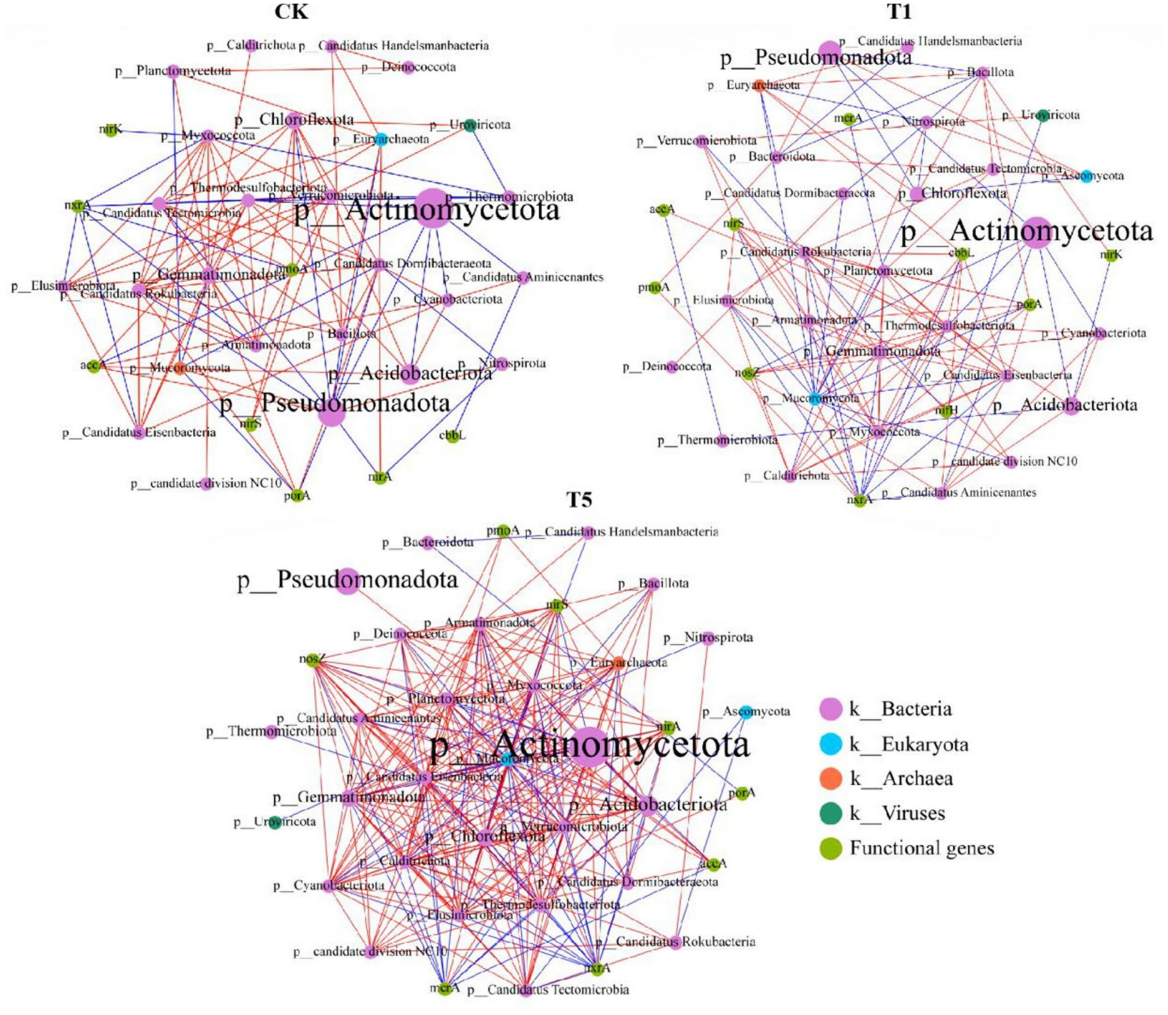
Network co-occurrence analysis of soil microbial communities that intercalated with C and N cycle genes in different treatments. A connection stands for a strong (Spearman’s *r* > 0.6) and significant (*p* < 0.05) correlation for the no treatment (CK) and mesotrione-treatments (T1 and T5). The red lines indicate a positive correlation between two species, while the blue lines represent a negative correlation. The color of each node is marked according to the kingdom of bacteria, archaea, eukaryotes, and viruses.

The effects of mesotrione on soil microbiology were analyzed by constructing a co-occurrence network model of soil microorganisms (top 30 phyla) associated with carbon and nitrogen cycle functional genes additional information follows (Figure 8). The total number of nodes, total number of edges, and average degree were higher in the T1 and T5 treatments compared to the control (Table 3). Compared to the control, the positive correlation ratio (%) decreased and the average path length increased in the T1 treatment, whereas the opposite occurred in the T5 treatment. These results indicate that mesotrione alters the network structure of soil microbial interactions, particularly among functional genes involved in the C and N cycling processes. The co-occurrence network analysis plots revealed that species nodes with higher relative abundance (at the phylum level) across all treatments exhibited fewer attached genes (e.g., Actinomycetota and Pseudomonadota), whereas species nodes with lower relative harbored a greater number of attached genes (e.g., Mucoromycota, Gemmatimonadota, Thermodesulfobacteriota, and Chloroflexota). Rare species within the soil microbial community were found to play equally important roles in both the N and C cycles. The number of attachment species increased at nodes of N and C cycle functional genes in the T1 and T5 treatments. It was shown that the microbial species which mediate the N and C cycles in the soil tended to increase under mesotrione stress.

**Table 3:** Topological indices of each network in Figure 8.

Network indexes	CK	T1	T5
Number of nodes	35	39	37
Number of edges	107	128	250
Positive correlation ratio (%)	68.22	64.84	73.20
negative correlation ratio (%)	31.78	35.16	26.80
Network diameter	6	6	6
Average path length	2.378	2.498	1.982
Average degree	6.114	6.564	13.514

## Discussion

### Effect of mesotrione on the physicochemical properties in soil

Soil serves as the foundation for plant growth, and its physicochemical properties were closely linked to the growth and development of plants. Among them, carbon, nitrogen, and phosphorus were essential elements required for plant growth (Agegnehu et al., 2016). The extensive use of chemical pesticides had led to changes in the soil physicochemical properties (Kekane et al., 2015). In this study, the total nitrogen, available phosphorus and organic matter in the soil were found to be significantly reduced, whereas soil pH was significantly increased during the experimental period (30–60 days) following mesotrione treatment. The result was similar to that of Cao et al. (2023), who reported the effects of mesosulfuron-methyl on the soil physicochemical properties, indicating that herbicide application can affect the soil physicochemical properties. Thus, soil microorganisms were sensitive to changes in soil environmental conditions, and chemical pesticide application can lead to alterations in soil microbial communities.

### Effects of mesotrione on the diversity of metabolic functions of soil microbial community

The Shannon, Simpson, McIntosh and Pielou indices, calculated based on soil microbial carbon metabolism data measured through Biolog-ECO microplate technology, provide a more accurate reflection of changes in the functional diversity, richness, and evenness of soil microbial communities (Zhao et al., 2020; Du et al., 2022; Wang et al., 2021). The results showed that soil microorganisms exhibited selective carbon source metabolism, and there were differences in metabolic activities among different types of carbon sources. The metabolic activity of soil microorganisms across multiple carbon sources declined under mesotrione stress, while the diversity, richness and evenness of soil microbial community functions showed reductions at different time periods. There were differences in the effects of different types of chemical pesticides on microbial metabolic functions. Sun et al. (2016) reported that a certain dose of mesotrione stimulated microbial activity and enhanced the diversity, evenness and richness of the microbial community. Zheng et al. (2013) demonstrated that low concentrations of organochlorine pesticides enhanced microbial physiological activity, whereas high concentrations inhibited such activity. There is a discrepancy between the results of this experiment and those reported in previous studies. The metabolic functions of microorganisms can be regulated in response to environmental changes, and this inconsistency may be attributed to variations in environmental factors.

### Effects of mesotrione on the structure of soil microbial community

A dynamic balance within soil microbial communities serves as a crucial indicator of the soil ecological health (Fierer et al., 2021). Studies have shown that the more complex the microbial community structure and the higher its diversity, the greater the ecological stability and the resilience against adverse environmental stresses (Toor et al., 2020). Soil microbial communities in natural environments are influenced by multiple factors, and the majority of scholarly opinion suggested that herbicide application alters the soil microbial community structure and reduced diversity (Chen et al., 2021). In this study, the diversity and richness of soil microbial communities decreased, and the community structure was altered from 1 to 60 days after mesotrione treatment, but gradually recovered over time, indicating the presence of a self-regulatory mechanism in soil microbiota to counter adverse environmental stresses. The responses of different microorganisms to different types of pesticides at different application doses vary (Widenfalk et al., 2008). In this study, the relative abundance of *Streptomyces*, *Gaiella* and *Nitrososphaera* increased, whereas that of *Agromyces*, *Luteitalea* and *Stenotrophomonas* decreased in the T1 treatment compared to the control. The relative abundance of *Nocardioides*, *Bradyrhizobium* and *Terrabacter* increased, while that of Var*iovorax* and *Desertimonas* decreased in the T5 treatment compared to the control. These results are similar to Rong et al. (2021), who investigated the effect of clomazone on microbial communities. Microorganisms exhibited selectivity toward pesticides, and certain species are capable of utilizing pesticides as nutrient sources for growth and reproduction (Parte et al., 2017). In the present study, we found that the relative abundance of *Nocardioides*, which are widely distributed in polar glaciers, alkaline soils, and heavy metal-contaminated soils, was significantly increased under the stress of high mesotrione-containing levels. The *Nocardioides* play important roles in environmental management, and *N. aromaticivorans* strains being effective in degrading dibenzofurans, and *N. nitrophenolicus* strains are capable of degrading p-Nitrophenol (Kubota et al., 2005; Yoon et al., 1999; Yoon et al., 2009). In agriculture, the widely used *Nocardioides* strain C190 strain was capable of metabolizing and producing triazine hydrolases that effectively degrade triazine herbicides (Topp et al., 2000). *Nocardioides* exhibited competitively advantages and proliferated more effectively under mesotrione stress compared to other genera. Therefore, we speculate that *Nocardioides* may metabolize mesotrione as a nutrient source and have the potential for application in its degradation; however, further experimental verification is still required.

### Effect of mesotrione on co-occurrence networks between soil microbiomes

The interaction patterns among various clusters within soil microbial communities are closely linked to their functional roles, with each microbial group contributing to a range of geobiochemical processes through complex interconnections (Kuypers et al., 2018). In this study, we found that the soil microbial interaction network underwent significant changes following mesotrione treatment, with low-relative-abundance microbial genera forming a highly connected modular core. A related study demonstrated that microorganisms with pesticide-degrading capacity tend to form high-complexity, symbiosis-associated clusters in soil networks under pesticide stress (Kuypers et al., 2018; Cao et al., 2023). In addition, this study further constructed the interaction network between major functional genes of the soil carbon (C) and nitrogen (N) cycles and the top 30 microbial clusters at the phylum level. The results revealed that phyla with low relative abundance exhibited higher connectivity with C and N cycle functional genes. Moreover, the microbial taxa mediating the connections of key functional genes involved in the C and N cycles showed an increasing trend under mesotrione stress. In summary, it is evident that genera with low relative abundance play equally important ecological roles in soil microbial communities. Key microbial taxa possessing degradation capabilities interact to form complex network modules which helps alleviate the stress imposed by chemical pesticides (Zhang et al., 2013; Kuypers et al., 2018).

### Effects of mesotrione on the abundance of functional genes for C and N cycle in the soil microorganisms

Soil microorganisms play a crucial role in soil carbon cycling through the decomposition of soil organic matter (Jiang et al., 2020). In this study, we found that the overall relative abundance of carbohydrase genes remained largely unchanged after mesotrione treatment, although variations were observed across different sampling periods. The results indicated that microorganisms exhibited diverse response mechanisms to mesotrione stimulation, and the activity of microbial taxa involved in carbohydrate metabolism was transiently enhanced. Soil microbes serve as key drivers in biogeochemical cycles, and their major functional genes involved in C and N cycling mediate the primary C (Gougoulias et al., 2014; Schimel and Schaeffer, 2012) and N (Kuypers et al., 2018) cycles in soils. In this study, we observed complex dynamic changes in soil microbial-mediated carbon and nitrogen cycling functional genes following mesotrione treatment. With regard to carbon cycling, the relative abundance of the *pmoA* gene was significantly reduced at 60 days in the T1 treatment, while the *accA* gene showed a significant increase at 30 days. In the T5 treatment, the *mcrA* gene exhibited a significant increase at 1 day, whereas the *accA* gene was significantly decreased at the same time point. Additionally, the *porA* gene displayed significantly increased abundance at both 10 and 60 days, and the *oorA* gene showed a significant increase at 30 days. These results are similar to Zhang et al. (2022), who reported the effect of thifluzamide on soil carbon cycle function. Among them, microbial clusters carrying the *accA*, *porA* and *oorA* genes were primarily involved in carbon fixation processes within the carbon cycle (Liu et al., 2021; Lynn et al., 2017), whereas those carrying the *pmoA* and *mcrA* genes were predominantly associated with methane metabolism processes in the carbon cycle (Huang et al., 2022). The above results suggested that under mesotrione stress, microbial taxa carrying functional genes potentially involved in the same carbon cycling process exhibit a complex complementary mechanism to maintain the overall carbon cycle in dynamic balance.

In terms of nitrogen cycling, the relative abundance of the *nirK* gene was significantly reduced at 1 day in the T1 treatment, while the *nirA* gene showed a significant decrease at 30 days. Additionally, the *nosZ* gene exhibited a significant increase at 30 days, and the *nxrA* gene was significantly reduced at both 30 and 60 days. In the T5 treatment, the *nirA* gene displayed a significant decrease at both 1 and 30 days, the *nosZ* gene showed a significant reduction at 1 and 10 days, the *nxrA* gene was significantly decreased at 20 days, and the *nifH* gene exhibited a significant increase at 30 days. This is similar to Cao et al. (2023), who reported the effect of mesosulfuron-methyl on functional genes of soil microbial nitrogen cycling. In particular, *nifH* is a functionally important gene involved in nitrogen fixation, *nxrA* plays a key role in nitrification, and *nirK*, *nirS*, and *nosZ* are critical genes associated with denitrification. Additionally, nirA is functionally significant in the assimilative nitrate reduction process (Wallenstein and Vilgalys, 2005; Zhang et al., 2013). Mesotrione exerts an inhibitory effect on microbial groups carrying the *nirK*, *nirA*, *nxrA*, and *nosZ* genes, while promoting those harboring the *nifH* gene. Therefore, it is hypothesized that mesotrione application may enhance nitrogen fixation by soil microorganisms.

## Conclusion

This study investigated the impacts of mesotrione on soil microbial communities and their functional roles. The results indicated that mesotrione treatment caused a short-term reduction in *α*-diversity, which gradually recovered over time. The soil microbial community structure exhibited complex temporal dynamics following exposure to mesotrione. Major functional genes involved in the same carbon cycling processes (e.g., carbon fixation pathways and methane metabolism) displayed complementary trends under mesotrione stress. Mesotrione exerted differential effects on key functional genes associated with various nitrogen cycling processes (e.g., assimilatory nitrate reduction, nitrogen fixation, nitrification, and denitrification), including the suppression of *nirA*, *nirK*, *nxrA*, and *nosZ* gene abundances and the enhancement of nifH abundance. Furthermore, low-abundance genera within the soil microbial community formed more complex and highly interconnected networks in response to mesotrione-induced stress. In summary, these findings contribute to a better understanding of how mesotrione influences soil microbial communities and their ecological functions.

## Data Availability

The datasets presented in this study can be found in online repositories. The names of the repository/repositories and accession number(s) can be found below: https://www.ncbi.nlm.nih.gov/, PRJNA1288065.
