# Supplementary material for: Mesotrione alters the structure of network interactions between soil microbes and affects C and N cycling functions
Source: Front Microbiol. 2026 Jan 2;16:1708771. doi: 10.3389/fmicb.2025.1708771 (PMC12807923; doi:10.3389/fmicb.2025.1708771)
Supplement: Supplementary file 1 [file Data_Sheet_1.docx]

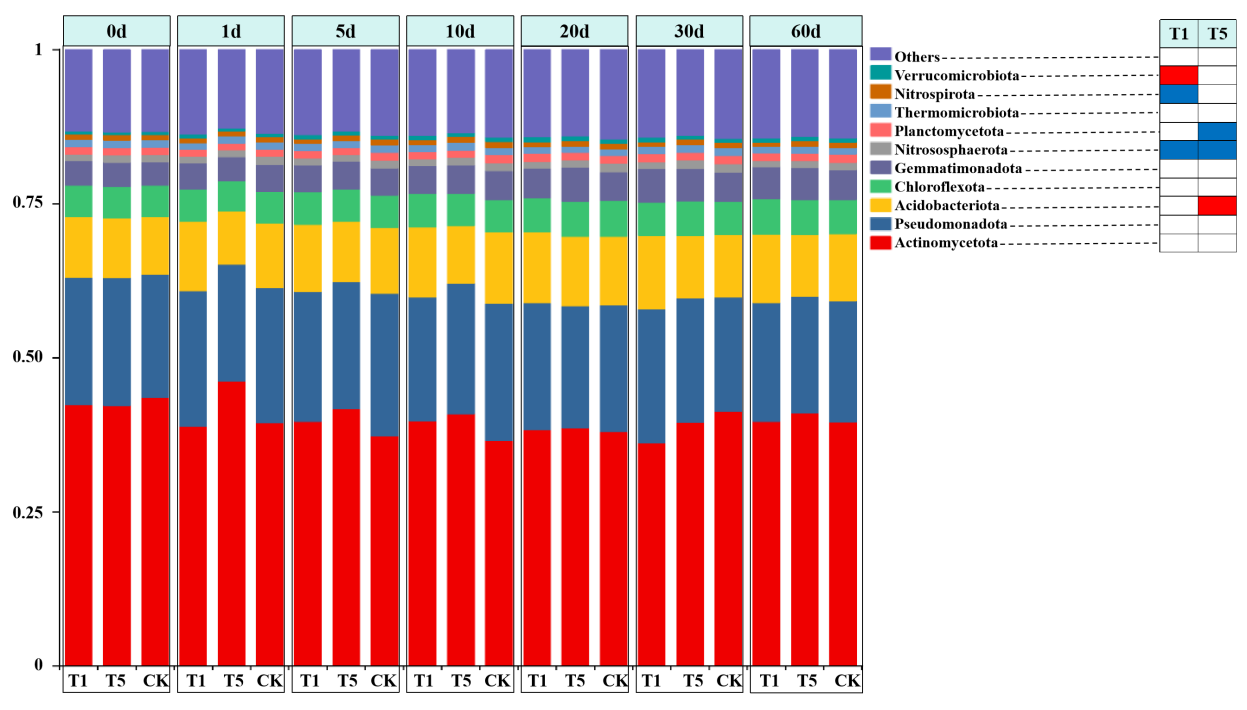


Fig. S1 Effects of mesotrione on soil microbial composition and community structure. The relative abundance of soil microorganisms(At the Phylums level, top10 ) in different treatments at different sampling periods was shown in Fig. A, and genera that showed a significant (P < 0.05) increased relative abundance after mesotrione-treated (red) or control (green) treatments were shown.


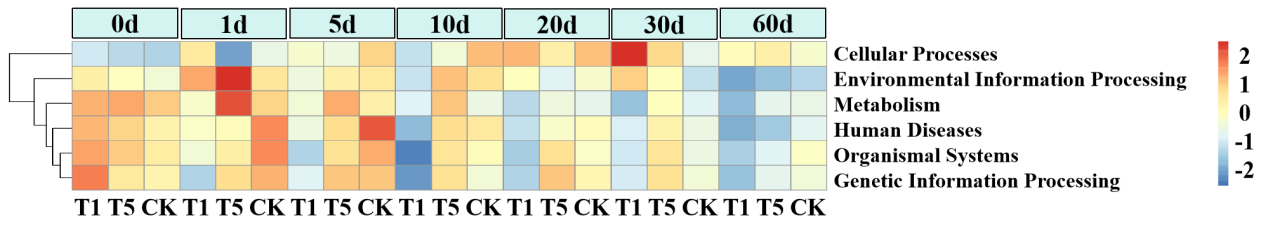


Fig. S2 Heat map shown the relative abundance (normalized and centered) of functional metabolic pathways (level 1) in different treatments. CK no mesotrione, T1: 180.00g a.i. hm^-2^, T5: 900.00 g a.i. hm^–2^.
